# Antimicrobial efficacy of disinfecting solutions against *S. mutans* on toothbrushes for school children in Uttar Pradesh, India

**DOI:** 10.6026/973206300223619

**Published:** 2026-06-30

**Authors:** Shivani Singh, Abhijeet Alok, Kamil Shahnawaz, Surender Kumar, Shantala Ramdas Naik, Kumar Piyush

**Affiliations:** 1 Department of Public Health Dentistry, Private Practitioner, Patna, Bihar, India; 2 Department of Oral Medicine and Radiology, Sarjug Dental College and Hospital, Darbhanga, Bihar, India; 3 Department of Dentistry, Darbhanga Medical College Hospital, Darbhanga, Bihar, India; 4 Department of Prosthodontics, Rajendra Institute of Medical Sciences, Ranchi, Jharkhand, India; 5 Department of Oral Medicine and Radiology, Rajendra Institute of Medical Sciences, Ranchi, Jharkhand, India; 6 Department of Conservative Dentistry and Endodontics, RKDF Dental College and Research Center, Bhopal, Madhya Pradesh, India

**Keywords:** Chlorhexidine gluconate (CHX), neem, *streptococcus mutans*, toothbrushes

## Abstract

Toothbrush bristles harbor pathogenic microorganisms like Streptococcus mutans, posing a continuous risk for reinfection and dental caries in children. Therefore, it is of interest to evaluate the ability of 5% neem, 5% green tea, 5% turmeric, 0.2% chlorhexidine (CHX) and tap water to disinfect Streptococcus mutans-contaminated toothbrushes after 12 hours of soaking. A randomized double-blind crossover study of 40 students (12-15 years) was conducted. All disinfectants significantly reduced bacterial counts compared to baseline, with the greatest reduction observed with 0.2% chlorhexidine, followed by 5% green tea. Thus, data shows that all disinfectants were effective, 0.2% chlorhexidine was the most effective and 5% green tea was a strong herbal alternative.

## Background:

Human body is home of many microorganisms. When a child is born, oral cavity is sterile, free of any microorganisms. *Streptococcus mutans (S. mutans)* is the most common organism that invades the oral cavity as the tooth erupts in oral cavity [1 , 2 3]. Oral hygiene practice had been followed all over world since 3000 BC. Various tools used were chewing sticks, neem twigs, bird feathers, animal bones, porcupine quills etc. Many methods have been used over a period of time to maintain oral hygiene. Toothbrushing is the most common method of maintaining oral hygiene. However, during use, toothbrushes can become contaminated with bacteria, saliva, blood and oral debris while removing plaque and food particles. As a result, contaminated toothbrushes may serve as a potential source of infection [4, 5, 6]. It is equally important to have tooth cleaning aids like toothbrush, dental floss etc. as much contamination free as possible [7]. In 1920, Cobb proposed that toothbrushes get contaminated after use and also said that repeated mouth infections may be due to contaminated toothbrush [8, 9]. Microorganisms can get entry into toothbrushes from either mouth or from surroundings like moist, humid conditions found in bathroom, two or more tooth brusher's bristles touching each other or many used toothbrushes kept in a closed container or collective use of toothbrush in low socioeconomic populations in developing and underdeveloped countries [10, 11]. Growth of microorganisms like *Pseudomonas aeruginosa* have been found in toothbrush covered with a cap after use [12]. Infection control is an important aspect which is now emphasized extensively in modern dentistry. Toothbrushes should be correctly stored, disinfected and changed at regular interval. Various solutions like chlorhexidine gluconate, sodium hypochlorite, 3 % neem, triclosan, distilled water etc. have been used as disinfecting solutions to reduce the contamination of toothbrush [13]. Therefore, it of interest to conduct the data on the antimicrobial efficacy of disinfecting solutions against *S. mutans* on toothbrushes of school children in Uttar Pradesh (India).

## Materials and Methods:

A crossover experimental trial with double blinding was conducted. There was a comparison within the group as well as between the groups. The study subjects and the statistician were blinded. The sample size was calculated using G*power v3.0.10, in which effect size of 0.40, an error of 0.20 and power (1-β err prob) of 0.80 was considered. A sample size of 40 participants was calculated. Out of 212 subjects, a total of 84 students were selected who had given informed consent and were eligible out of which 40 were randomly selected by lottery method. The study was completed in one month. The study was commenced as an *in vitro* study. The study started with distribution of anchor toothbrushes to all the subjects and all subjects were asked to brush once daily for five consecutive days. New toothbrushes were given to all the participants every fifth day. Subjects were asked to brush from the new toothbrush. Upon the completion of the fifth day, all subjects received brand-new toothbrushes. Subjects were advised to brush from this new toothbrush (which was given to them on 5th day) from next day i.e., 6th day. Subjects used to brush from this new tooth brush for next 5 days and they were again given new tooth brush at the end of 5 days and so on. The used toothbrushes were collected and placed in the sterilized glass bottles containing brain heart infusion broth. At the end of first five days (for baseline) and various other disinfecting solutions afterwards (after every five days) were transferred to microbiology laboratory for assessment of *S. mutans* count. In *in vitro* stage, the toothbrushes were placed in brain heart infusion broth for baseline and disinfecting solutions and were removed after 12 hours respectively. Turbidity was matched for baseline sample with McFarland turbidity standard tubes No.1. Sub culture was done on Mitis- Salivarius agar by pour plate method and then incubated at 37 degree Celsius for overnight duration after which CPUs were counted. Five solutions were categorized into group (GRP) I having only distilled water, group II consists of 5% neem, group III comprising of 5% turmeric, group IV (5% green tea) and group V included 0.2 % chlorhexidine solution (Figure 1A, Figure 1B, Figure 1C, Figure 1D, Figure 1E, Figure 1F - see PDF). Forty toothbrushes were analyzed in each group. The study was approved by the Institutional ethical committee (IDS/ETHCC/2016/37). The parents of the children who agreed to allow their child to participate in the study signed an informed consent form after receiving a participant information letter. The participant inclusion criteria were: children between 12-15 years of age who were cooperative,comprising of two multirooted teeth and three single rooted teeth in each quadrant, free from any systemic diseases. The exclusion criteria were: 1) children undergoing any dental treatment and orthodontic treatment at the time of the study, 2) children using any mouth rinse or any antibiotics or fluoride in any form of oral hygiene aids since three months or during the study. Microbial analysis was done in MDC, Diagnostic centre, Bareilly (U.P), India. For baseline data, autoclaved brain heart infusion broth was used as a medium. Turbidity of the baseline sample was matched by McFarland turbidity tube No. 1. For study samples, after immersing in the specific disinfecting solution the sample was incubated at 37 degree celsius for overnight duration after which subculture was done on mitis- salivarius agar by pour plate method and incubated at 37 degree Celsius for overnight duration. Then colony forming units (CFUs) were counted.

## Statistical analysis:

The collected data were transferred to Microsoft Excel and analyzed using the statistical package for social sciences (SPSS IBM v21.Inc. Chicago. IL) software. For intragroup comparison, Wilcoxon signed rank test was employed while the Friedman and chi-square tests were applied for intergroup comparison.

## Results:

Forty individuals participated in the investigation. Gender wise distribution showed 14 (35%) girls and 26 (65%) boys participated in this study. All the subjects had to undergo each phase of the study procedure phase I (group I; distilled water), phase II (group II; 5% neem), phase III (group III; 5% turmeric), phase IV (group IV; 5% green tea), phase V (group V; 0.2% chlorhexidine). There were no drop-outs in any group. The age group of subjects ranged from 12-15 years. 20% subjects belonged to 12 and 15 years age group and 30% subjects belonged to 13 and 14 years of age groups. The data is CFU which is an exponential data, which cannot be analyzed as any other data. So, a log transformation of the CFU was done. All the mean values shown in the tables are after log transformation. Statistical analysis showed that the intragroup comparison of baseline, water, 5% neem, 5% turmeric, 5% green tea, 0.2% chlorhexidine as disinfecting solution was highly significant (<0.001) (Table 1). *S. mutans* level was high at baseline but slighty less in water. The levels of *S. mutans* were continuously reducing in 5% neem, 5% turmeric and 5% green tea. There was no growth of *S. mutans* in 0.2% CHX. Comparison of *S. mutans* level between each group was done. When compared to baseline, group I (water) was not statistically significant. The reduction of *S. mutans* was very less in group I (water) as disinfecting solution. When group II (5% neem), III (5% turmeric) and IV (5% green tea) were compared between themselves and at baseline, the result was statistically significant. Group V (0.2% chlorhexidine) shows no bacterial growth (Table 2). There was reduction of *S. mutans* CFU with baseline solution. Reduction of CFU was highest in 0.2% chlorhexidine (5.2) followed by 5 % green tea (3.3), 5% turmeric (2.3), 5% neem (1.6). The lowest mean reduction CFU was at water (0.175) (Figure 2 - see PDF).

## Discussion:

Microbiological contamination of the oral cavity has been a widely discussed topic in various literatures. Very less attention has been paid on the contamination of toothbrushes. Few studies have shown that toothbrushes act as a vehicle in microbial contamination if not properly disinfected. Regular changing of toothbrushes in short span of time is not advisable as it will be an expensive way of prevention of microbial transfer [14, 15, 16]. Various disinfecting solutions are there which should be used to disinfect toothbrushes. The main transmissible etiological agent of dental caries is *S. mutans*. The risk of reinfection of teeth because of *S. mutans* on toothbrushes makes it relevant to investigate the methods of toothbrush care [17]. The presence of *S. mutans* CFUs on toothbrushes has been confirmed by many studies as seen in our study [18, 19]. A study conducted by Taji & Rogers did not detect *S. mutans* on any toothbrushes which may be due to small sample size [20]. The mean reduction of *S. mutans* CFU with baseline was highest in 0.2% chlorhexidine (5.2) followed by green tea (3.3), 5% turmeric (2.3), 5% neem (1.6). The lowest mean reduction CFU was at water (0.175). A cationic agent exhibiting a broad-spectrum antimicrobial effect, chlorhexidine has proven to be an effective antimicrobial solution in a number of studies. Study conducted by Grewal and Swaranjit and Jesi Chandrika *et al.* showed that 0.2 % chlorhexidine can inhibit *S. mutans* count effectively as seen in our study [21, 22]. Different children may differ in microbial contamination depending on effectiveness of their tooth brushing technique. Neem has been used in India from ancient times because of its medicinal importance. Neem is economical and is most commonly available in rural areas. Polyphenolic tannins found in neem extract bind to surface-associated proteins produced by bacteria, causing bacterial aggregation and a decrease in glucosyl transferase activity, which lowers the number of *S. mutans.* 3% neem was used in study conducted by Balappanavar *et al.* whereas in our study, 5 % neem was used as antimicrobial solution [23]. This is the only study which used green tea as disinfecting solutions. Antimicrobial efficacy of green tea was more than turmeric and neem. This study was done to check the efficacy of various disinfecting solutions. Toothbrushes were not used continuously as after every 5th day; a new tooth brush was given to each and every subject. Tooth brushes dipped in disinfecting solutions were sent to microbiological investigation. The limitations of this study were that it did not consider all the microorganisms present on toothbrushes and that only one soaking regimen was used. Further studies are required to check for the efficacy of various antimicrobial solutions at different concentrations on a larger sample size.

## Conclusion:

We show that toothbrush contamination is common and that disinfection can help prevent infection transmission. Among the tested agents, 0.2% chlorhexidine was the most effective disinfectant, while green tea proved to be a safe, accessible and effective herbal alternative. Data support the use of both chemical and herbal disinfectants for maintaining oral hygiene and reducing toothbrush contamination.

## Figures and Tables

**Table 1:** Intra group comparison of baseline to each group

	**N**	**Mean**	**Std. Deviation**	**Median**	**Mean rank**	**P-value**
Baseline	40	5.275	1.826	5	5.49	<0.001*
Water	40	5.1	1.614	5	5.33	
5% Neem	40	3.625	1.334	4	3.84	
5% Turmeric	40	2.9	1.128	3	3.21	
5% Green Tea	40	1.75	0.84	2	2.14	
0.2% CHX	40	0	0	0	1	
*denotes statistically
significant using Friedman
Test, Chi-square- 189.324

**Table 2:** The inter group comparison between each group.

	**N**	**Mean**	**Std. Deviation**	**Median**	**Water Log**	**5% Neem Log**	**5% Turmeric Log**	**5% Green Tea Log**	**0.2% CHX**
Baseline	40	5.275	1.826	5	0.020*	<0.001*	<0.001*	<0.001*	<0.001*
Water	40	5.1	1.614	5	-	<0.001*	<0.001*	<0.001*	<0.001*
5% Neem	40	3.625	1.334	4	-	-	<0.001*	<0.001*	<0.001*
5% Turmeric	40	2.9	1.128	3	-	-	-	<0.001*	<0.001*
5% Green Tea	40	1.75	0.84	2	-	-	-	-	<0.001*
0.2% CHX	40	0	0	0	-	-	-	-	-
